# Differentially expressed lncRNAs in liver tissues of TX mice with hepatolenticular degeneration

**DOI:** 10.1038/s41598-020-80635-0

**Published:** 2021-01-14

**Authors:** Juan Zhang, Ying Ma, Daojun Xie, Yuancheng Bao, Wenming Yang, Han Wang, Huaizhou Jiang, Hui Han, Ting Dong

**Affiliations:** 1 Encephalopathy Center, the First Affiliated Hospital of Anhui University of Chinese Medicine, No 117 Meishan Road, Shushan District, Hefei, 230031 People’s Republic of China; 2 Basic Department of Traditional Chinese Medicine, Anhui University of Chinese Medicine, No 1 Qianjiang Road, Xinzhan District, Hefei, 230012 People’s Republic of China; 3 Graduate School, Anhui University of Chinese Medicine, No 1 Qianjiang Road, Xinzhan District, Hefei, 230012 People’s Republic of China

**Keywords:** Neuroscience, Neurology

## Abstract

Wilson's Disease (WD), an ATP7B-mutated inherited disease that affects copper transport, is characterised by liver and nervous system manifestations. Long non-coding (ln-c) RNAs are widely involved in almost all physiological and pathological processes in the body, and are associated with numerous diseases. The present study aimed to elucidate the lncRNA-mRNA regulation network in a TX WD mouse model using RNA sequencing (RNA-seq). lncRNA expression profiles were screened using RNA-seq and real-time polymerase chain reaction, and differentially expressed lncRNAs and mRNAs were identified. To analyse the biological functions and pathways for the differentially expressed mRNAs, gene ontology and pathway enrichment analyses were performed. A significantly correlated lncRNA-mRNA relationship pair was calculated by CNC analysis to construct differential lncRNA and mRNA co-expression networks. A total of 2564 significantly up-regulated and 1052 down-regulated lncRNAs, and 1576 up-regulated and 297 down-regulated mRNAs, were identified. These genes were found to be associated with key processes such as apoptosis, and KEGG analysis revealed enrichment in the drug metabolism-cytochrome P450 pathway, PPAR signalling pathway, Notch signalling pathway, and MAPK signalling pathway. The identified differential lncRNAs may be involved in the pathogenesis and development of WD liver injury.

## Introduction

Wilson's disease (WD), also known as hepatolenticular degeneration, is an autosomal recessive disorder of copper metabolism caused by an ATP7B gene mutation^1^. WD results in a decrease in copper excretion in bile, which leads to the accumulation of copper in various organs, including the liver and brain, causing liver, and nerve damage, and mental symptoms^2^. While the clinical manifestations of WD patients involve multiple systems, liver disease is most prevalent, and is more common in younger children. WD patients begin to accumulate copper in the liver from birth; hence, most patients initially present with liver cirrhosis.

As WD is an autosomal recessive single-gene genetic disease, disease prevention and treatment based on the pathogenesis can be investigated. Long non-coding (Inc)RNAs were once considered by-products of the transcription process, and the “noise” of gene transcription with no biological functions^3^. However, it has been demonstrated that lnc-RNAs are widely involved in almost all physiological and pathological processes in the body, and are associated with the occurrence and development of many diseases^4^. Copper ions have a strong ability to produce free radicals, making excess copper potentially toxic. The main mechanism leading to liver fibrosis or cirrhosis in WD is through hepatic stellate cells (HSCs), which become activated by various fibrogenic pathways, and cause an imbalance in extracellular matrix (ECM) synthesis and degradation during repair of liver injury. HSCs are the main effectors of hepatic fibrosis. A variety of lncRNAs have been found to play important regulatory roles in the activation of HSCs^5^, and are suggested to have a prominent role in hepatic fibrosis in WD; thus, they may serve as predictive markers or therapeutic targets for disease occurrence.

In this study, lncRNA expression profiles in liver tissues of TX WD mice were assessed using RNA-seq, to investigate the mechanism of lncRNA involvement in WD liver injury further.

## Results

### Liver histopathology

**Figure 1:**
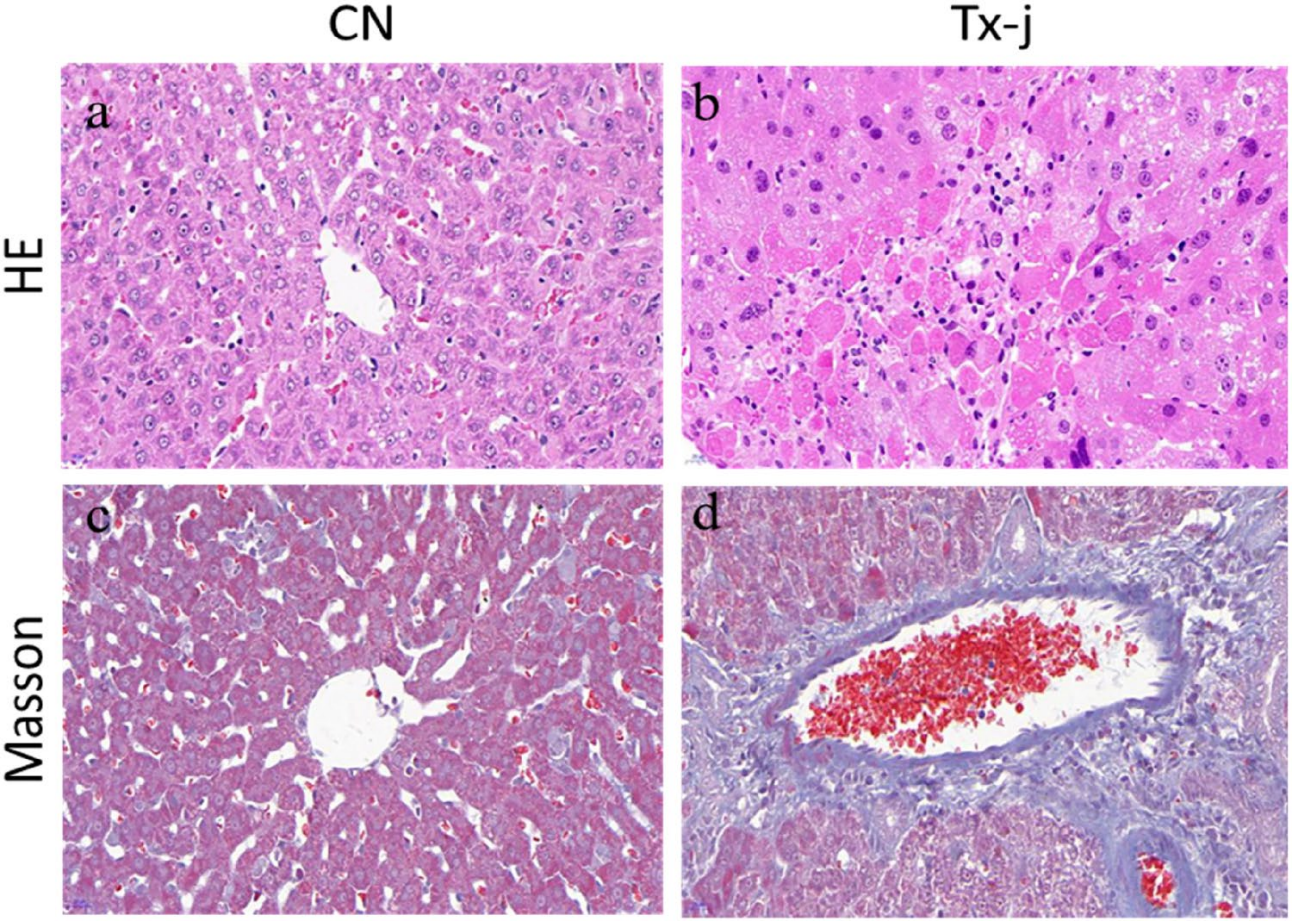
Haematoxylin and eosin staining in the control group showed clearly structured hepatic lobules, and the hepatocytes were stationary in the centre; veins radiated throughout the tissue, and the central veins, the arteriovenous structure, and the bile duct appeared normal. In the model group, there was extensive necrosis of hepatocytes and normal hepatic lobule structure disappeared; a large amount of inflammatory cell infiltration was evident. Masson staining showed that, in the control group, only a few collagen fibres were found in the manifold area and central vein. The liver tissue of the model group had extensive hyperplasia, extending from the manifold area to the surrounding area, forming a complete pseudolobular structure of different sizes.

Haematoxylin and eosin staining in the control group showed clearly structured hepatic lobules, and the hepatocytes were stationary in the centre; veins radiated throughout the tissue, and the central veins, the arteriovenous structure, and the bile duct appeared normal. In the model group, there was extensive necrosis of hepatocytes, and normal hepatic lobule structure disappeared; a large amount of inflammatory cell infiltration was evident. Masson staining showed that, in the control group, only a few collagen fibres were found in the manifold area and central vein. The liver tissue of the model group had extensive hyperplasia, extending from the manifold area to the surrounding area, forming a complete pseudolobular structure of different sizes (Fig. 1).

### Expression profile of lncRNAs in the liver

**Figure 2:**
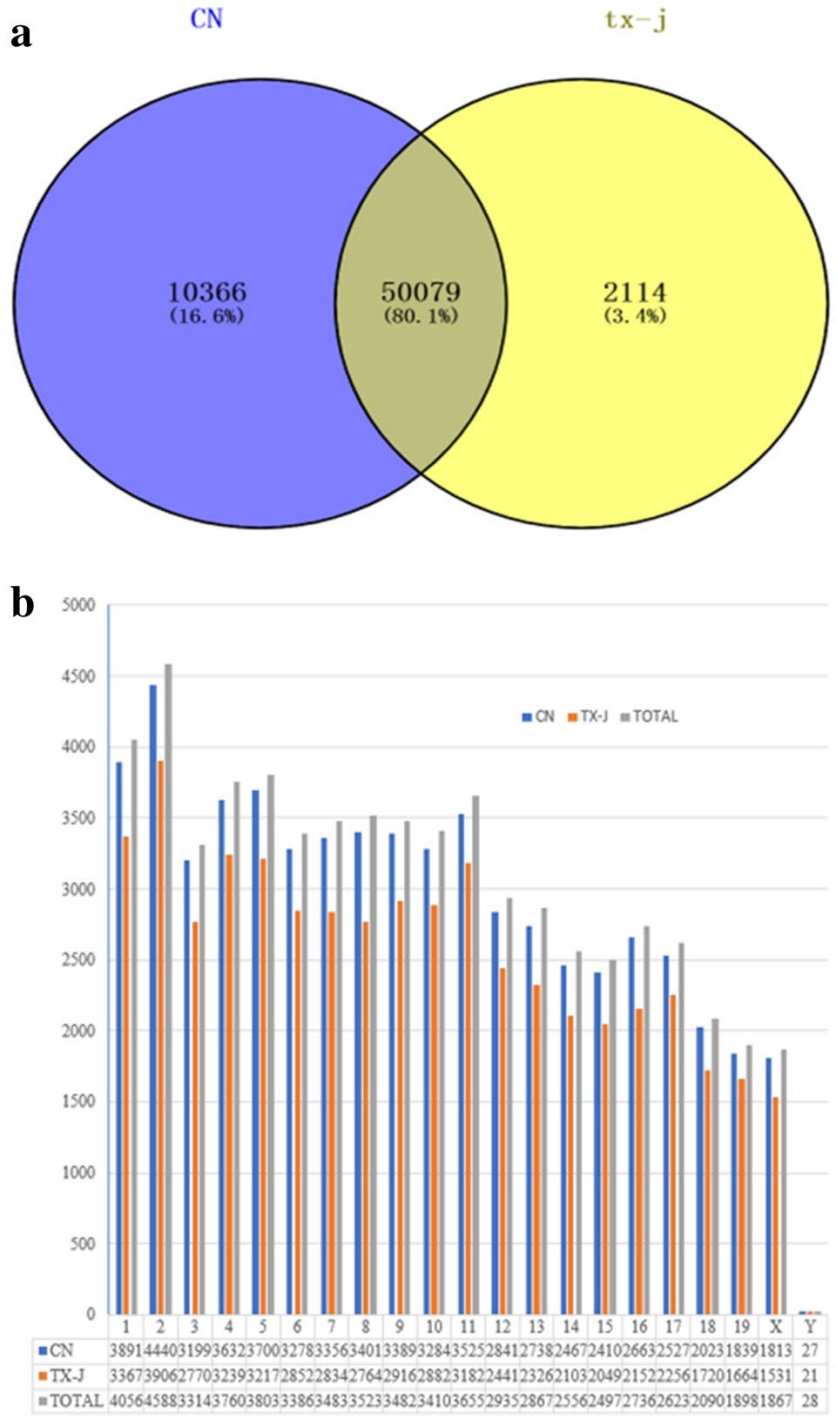
(**a**) In total, we identified 62,559 lncRNA transcripts in which fragments per kilobase of exon per million reads mapped (FPKM) were above 0 among any one of 4 CN samples or 4 tx-j samples; further, 50,079 lncRNAs were expressed in both groups in which the FPKM was above 0 among any one of 4 CN samples and 4 tx-j samples (**a**). (**b**) The most common type of lncRNAs was intergenic, the next were intronic sense, exonic sense and exonic antisense, and intronic antisense was the least type. In addition, we analysed the distribution of identified lncRNAs on the mouse chromosomes; the 62,559 lncRNA transcripts could be found in all chromosomes, including ChrX and ChrY, and chromosome 2 included the most lncRNAs (**b**). Almost all chromosomes (excluding Chr19, ChrX, and ChrY) could generate more than 2000 lncRNA transcripts (**b**).

We characterised the lncRNA expression profile by performing deep RNA-seq experiments on 4 tx-j and 4 control mouse liver tissues. In total, we identified 62,559 lncRNA transcripts in which fragments per kilobase of exon per million reads mapped (FPKM) were above 0 among any one of 4 CN samples or 4 tx-j samples; further, 50,079 lncRNAs were expressed in both groups in which the FPKM was above 0 among any one of 4 CN samples and 4 tx-j samples (Fig. 2a). The most common type of lncRNAs was intergenic, the next were intronic sense, exonic sense and exonic antisense, and intronic antisense was the least type. In addition, we analysed the distribution of identified lncRNAs on the mouse chromosomes; the 62,559 lncRNA transcripts could be found in all chromosomes, including ChrX and ChrY, and chromosome 2 included the most lncRNAs (Fig. 2b). Almost all chromosomes (excluding Chr19, ChrX, and ChrY) could generate more than 2000 lncRNA transcripts (Fig. 2b).

### Identification of differentially expressed lncRNAs and protein-coding genes

**Figure 3:**
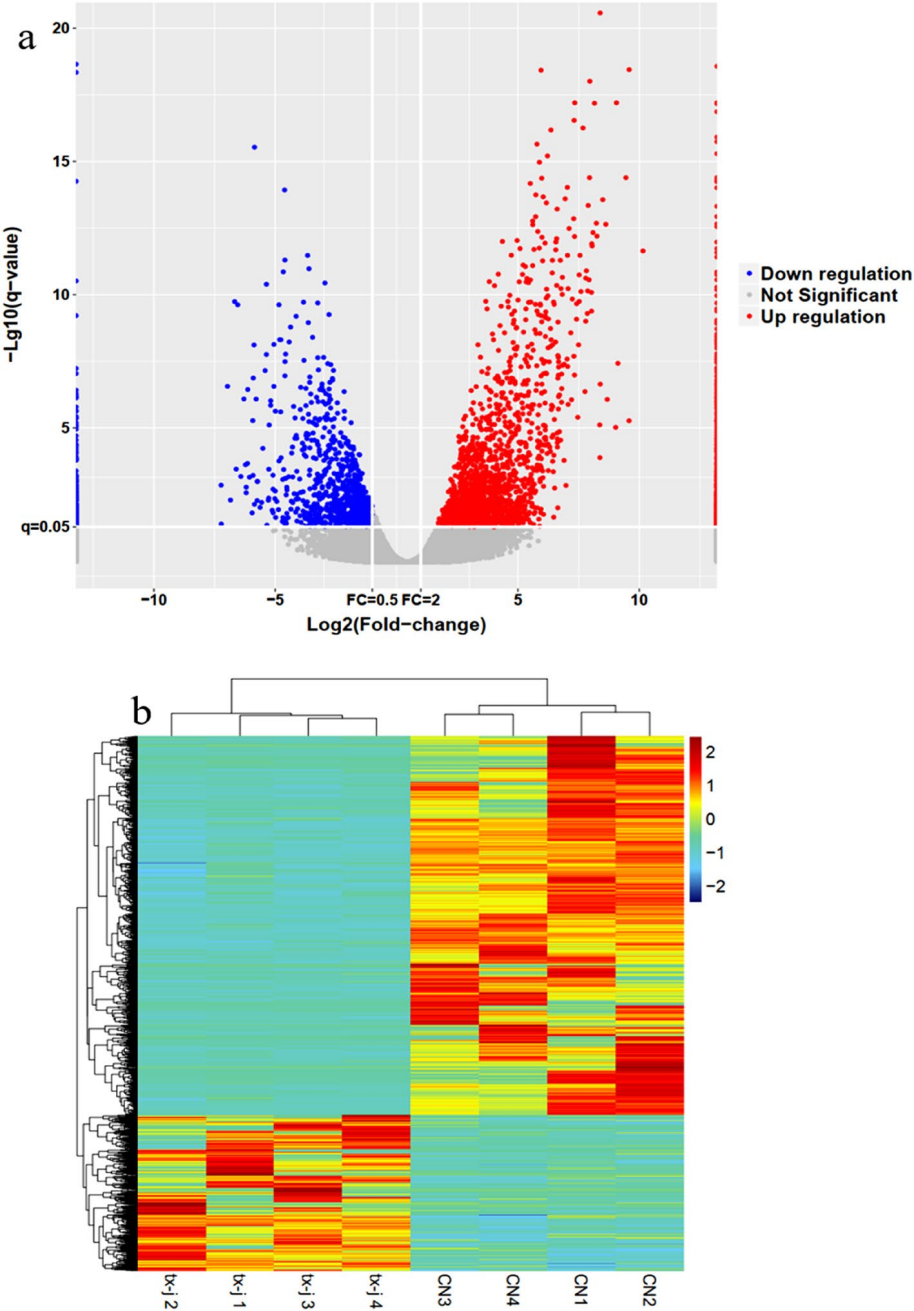
(**a**,**b**) Display the lncRNAs’ expression by use of volcano plots and heatmap. Supervised hierarchical cluster analysis shows that the different mRNA can correctly distinguish the model group from the control group.

Intending to explore the role of lncRNAs in the tx-j model, we performed RNA-seq to obtain the expression profiles of lncRNAs. As a result, there were 3616 lncRNA transcripts in the control and model groups. Thereinto, 2564 up-regulated and 1052 down-regulated lncRNAs were proofed by fold change > 2, P < 0.05. Figure 3a,b (Pheatmap-1.0.12,URL: https://i.loli.net/2020/11/05/Z6cyuBk1oEvX8AP.png) display the expression of lncRNA using volcano plots and heatmap. Supervised hierarchical cluster analysis shows that the different mRNA can correctly distinguish the model group from the control group (Fig. 3b). Further, we chose FPKM values greater than 1 for further analysis. In Table 2, the top 10 up-regulated and down-regulated known lncRNAs are scheduled.

Additionally, 1873 protein-coding genes were found dysregulated in TX mice by twofold, of which 1576 protein-coding genes were up-regulated and 297 protein-coding genes down-regulated.

### Construction of lncRNA-mRNA co-expression network and function prediction

**Figure 4:**
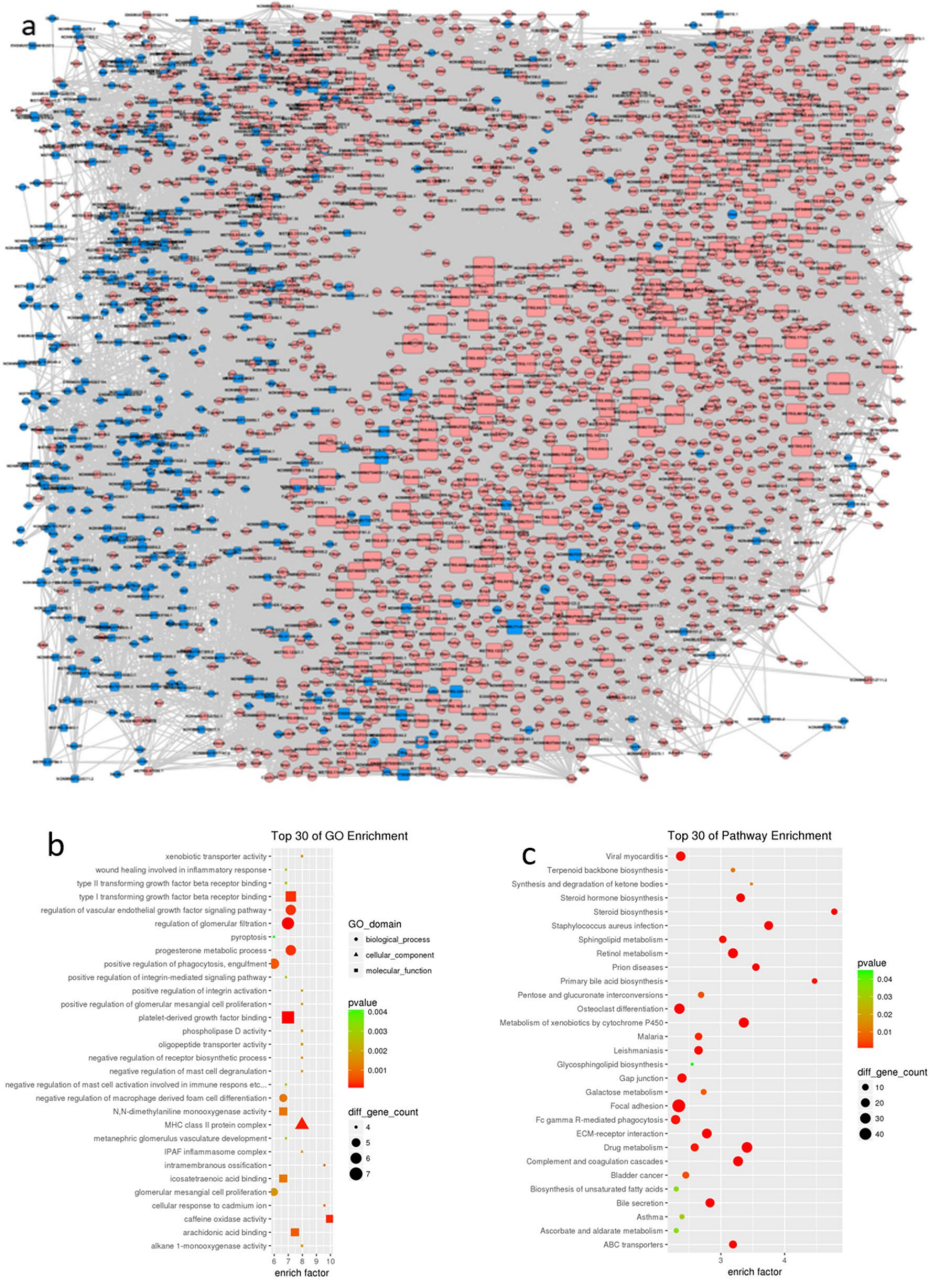
(**a**) After selecting the Pearson’ s correlation coefficients of these lncRNAs and mRNAs equal to or > 0.98 (Table 1), a network is founded in each of the aforementioned groups using the Cytoscape program. The protein-coding genes belonging to the whole lncRNA-mRNA co-expression network were enriched with GO and pathway analysis, and the top 30 terms were presented (**b**,**c**).

After selecting the Pearson’s correlation coefficients of these lncRNAs and mRNAs equal to or > 0.98 (Table 1), a network was founded in each of the groups mentioned above using the Cytoscape program (Fig. 4a). More than 198 lncRNAs could connect with more than 100 protein-coding genes, and almost all of those were up-regulated, excluding 6 lncRNAs. Meanwhile, many protein-coding genes could connect with dozens of lncRNAs. Furthermore, the details of the top10 connections of up- and down-regulated DELs and protein-coding genes in lncRNA-mRNA co-expression network are listed in Table 2. The protein-coding genes belonging to the whole lncRNA-mRNA co-expression network were enriched with GO and pathway analysis (Table 1), and the top 30 terms were presented (Fig. 4b,c).

**Table 1:** The details of qPCR primers.

lncRNA_id	Forward primer	Product length (bp)	Fold change/p-vale (tx-j vs CN, qPCR)
NONMMUT149595.1	Forward: AGCACGAAGCTGAAGGCGTC	171	0.47 **
	Reverse: GCTCCCTGGCCTGGAGGATTG		
NONMMUT099727.1	Forward: CCCCAAGCCTTGCTCAGGGT	151	0.35 **
	Reverse: CGTCCACAGTTCCGAGACTGGC		
ENSMUST00000129245	Forward: AGTGCCCTAGTGAGGGGGCA	146	12.3 **
	Reverse: CAAGGGACGACCCTCCTCGG		
ENSMUST00000150851	Forward: CGTCCTTCCCTGGTGGGTGT	175	13.5 **
	Reverse: GGGCAGCTCGTGGAACCTGA		
ENSMUST00000136359	Forward: CGTGGCGGCTGGTCGGATAA	106	24.7 **
	Reverse: CAGACGGCGGTCCTCACCTG		
ENSMUST00000181536	Forward: TTTGGCTACCCGCCCCTTGC	115	9.87 **
	Reverse: CAGGCCTAGCAGACGCCACG		
Beta-actin	Forward: CCTCACTGTCCACCTTCC	120	–
	Reverse: GGGTGTAAAACGCAGCTC		

**Means p < 0.01.

**Table 2:** Top10 upregulated and down-regulated DELs (FPKM > 1) between tx-j and control mice.

lncRNA_id	Locus	Length	tx-j	CN	log2FC	P value	Updown
NONMMUT060008.2	7:14,410,690–14,411,717	1028	0.172	172.514	− 9.973	2.52E−41	DOWN
NONMMUT060075.2	7:16,915,382–16,916,322	941	0.067	27.846	− 8.690	2.63E−38	DOWN
NONMMUT096375.1	15:89,767,563–89,768,777	817	0.057	6.597	− 6.844	1.33E−04	DOWN
NONMMUT152860.1	8:105,051,546–105,058,411	1236	0.037	3.502	− 6.546	5.83E−13	DOWN
NONMMUT008761.2	11:20,225,237–20,226,853	1617	0.206	17.552	− 6.414	1.12E−05	DOWN
NONMMUT001029.2	1:58,405,414–58,407,353	1940	0.123	9.000	− 6.196	6.17E−05	DOWN
NONMMUT050970.2	4:155,619,936–155,623,338	3403	0.132	9.308	− 6.143	3.01E−06	DOWN
NONMMUT027553.2	16:87,376,654–87,378,943	2290	0.066	3.928	− 5.903	6.59E−10	DOWN
MSTRG.41348.1	4:61,444,453–61,524,270	560	0.044	2.549	− 5.872	2.77E−11	DOWN
NONMMUT054921.2	5:137,336,283–137,338,185	1176	0.052	3.016	− 5.852	1.59E−19	DOWN
MSTRG.12342.1	12:23,832,607–23,841,289	428	23.898	0.046	9.031	8.40E−08	UP
MSTRG.64316.1	X:33,549,148–33,863,315	1414	3.692	0.007	9.060	2.41E−21	UP
MSTRG.36679.1	3:12,833,226–12,849,812	1630	3.330	0.006	9.117	1.60E−10	UP
NONMMUT089191.1	13:3,386,160–3,388,230	2071	5.325	0.009	9.139	3.52E−27	UP
MSTRG.64312.1	X:33,541,135–33,841,298	643	20.056	0.030	9.365	1.20E−25	UP
MSTRG.40589.1	4:40,651,444–40,660,004	297	22.185	0.032	9.448	2.50E−18	UP
MSTRG.47803.6	5:145,463,518–145,800,965	1195	12.340	0.016	9.584	1.03E−22	UP
NONMMUT050450.2	4:144,131,848–144,133,881	2034	59.925	0.053	10.152	3.13E−15	UP
NONMMUT089188.1	13:3,363,013–3,363,773	761	14.982	0.012	10.239	1.44E−29	UP
MSTRG.61463.4	9:58,702,406–58,712,258	1161	28.375	0.017	10.718	2.71E−31	UP

**Figure 5:**
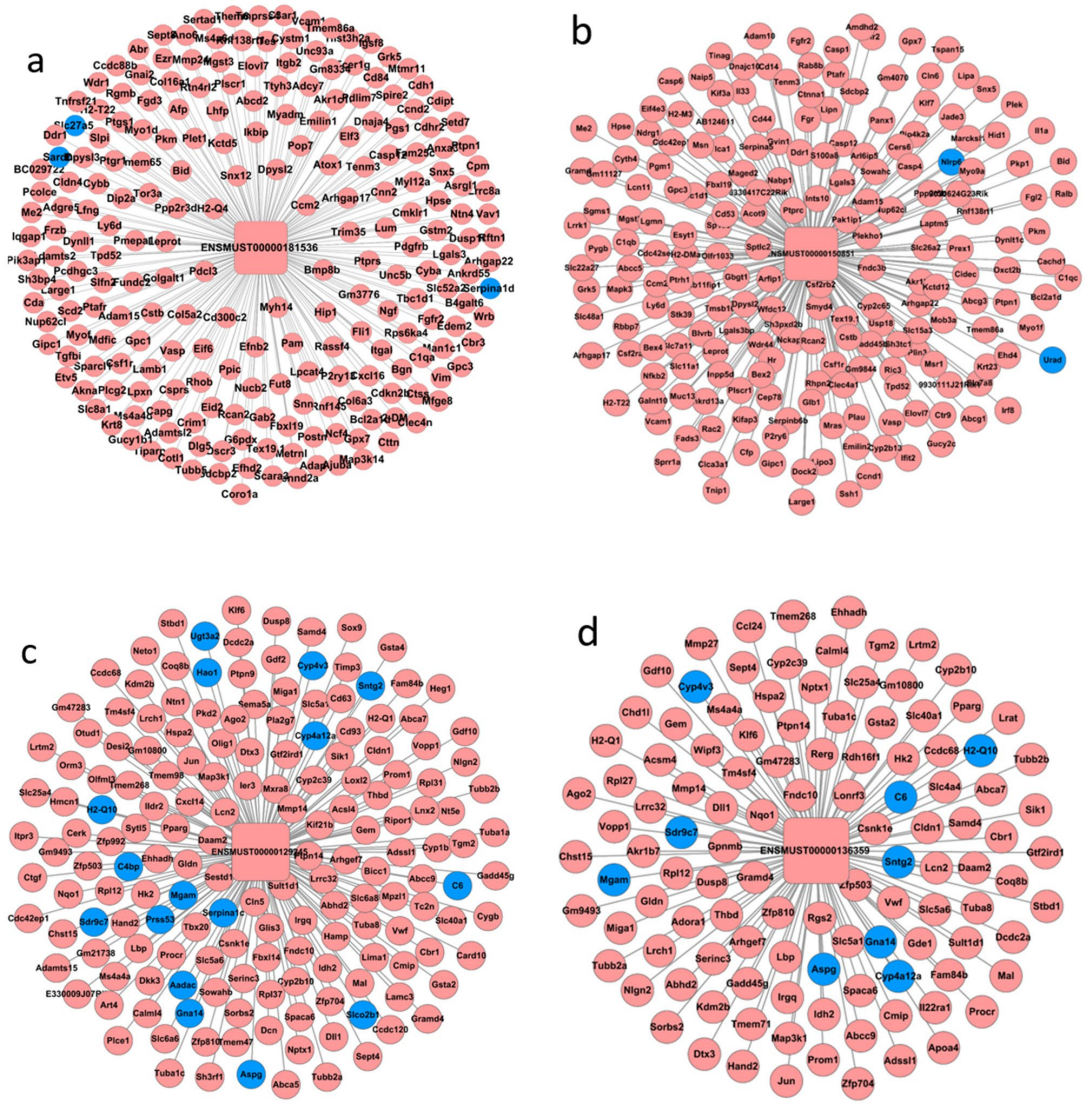
As shown in (**a**), ENSMUST00000181536 (Snhg19) correlated with 222 protein-coding genes, and the enrichment analysis showed that Snhg18 may be related to leukocyte transendothelial migration, phagosome, glutathione metabolism, regulation of actin cytoskeleton, natural killer cell mediated cytotoxicity, focal adhesion, Fc epsilon RI signalling pathway, cell adhesion molecules, apoptosis, and Fc gamma R-mediated phagocytosis. As shown in (**b**), ENSMUST00000150851 (Meg3) correlated with 200 protein-coding genes, and KEGG enrichment analysis pointed out that it may participate in sphingolipid metabolism, regulation of actin cytoskeleton, glycosaminoglycan degradation, complement and coagulation cascades, adherens junction, leukocyte transendothelial migration, galactose metabolism, haematopoietic cell lineage, chemokine signalling pathway, p53 signalling pathway, B cell receptor signalling pathway, Fc epsilon RI signalling pathway, ABC transporters, mitogenactivated protein kinase (MAPK) signalling pathway, and apoptosis. As shown in (**c**), ENSMUST00000129245 (Meg3) correlated with 169 protein-coding genes, that enriched to dozens of KEGG pathway, such as gap junction, ABC transporters, peroxisome, glutathione metabolism, GnRH signalling pathway, complement and coagulation cascades, peroxisome proliferator-activated receptor (PPAR) signalling pathway, metabolism of xenobiotics by cytochrome P450, phagosome, tryptophan metabolism, fatty acid degradation, carbohydrate digestion and absorption, Notch signalling pathway, ribosome, and toll-like receptor signalling pathway. KEGG analysis revealed that two lncRNA transcripts were both related to ABC transporters and complement and coagulation cascades, although these two transcripts did not share the same gene of interaction. As shown in (**d**), ENSMUST00000136359 (H19) correlated with 169 protein-coding genes, including vitamin digestion and absorption, galactose metabolism, carbohydrate digestion and absorption, retinol metabolism, phagosome, gap junction, arachidonic acid metabolism, butanoate metabolism, complement and coagulation cascades, antigen processing and presentation, PPAR signalling pathway, ABC transporters, Notch signalling pathway, bile secretion, and MAPK signalling pathway.

Certain established lncRNAs that could connect with more than 100 protein-coding genes were identified, such as Meg3(ENSMUST00000129245 and ENSMUST00000150851), H19(ENSMUST00000136359), and Snhg18 (ENSMUST00000181536). Next, the sub-network of four lncRNA transcripts were visualised with Cytoscape (Fig. 5), and GO enrichment and KEGG^6,7^ pathway analysis were also performed with cluster Profiler (Table 2). As shown in Fig. 5a, ENSMUST00000181536 (Snhg19) correlated with 222 protein-coding genes, and the enrichment analysis showed that Snhg18 might be related to leukocyte transendothelial migration, phagosome, glutathione metabolism, regulation of actin cytoskeleton, natural killer cell-mediated cytotoxicity, focal adhesion, Fc epsilon RI signalling pathway, cell adhesion molecules, apoptosis, and Fc gamma R-mediated phagocytosis. As shown in Fig. 5b, ENSMUST00000150851 (Meg3) correlated with 200 protein-coding genes, and KEGG enrichment analysis pointed out that it may participate in sphingolipid metabolism, regulation of actin cytoskeleton, glycosaminoglycan degradation, complement and coagulation cascades, adherens junction, leukocyte transendothelial migration, galactose metabolism, haematopoietic cell lineage, chemokine signalling pathway, p53 signalling pathway, B cell receptor signalling pathway, Fc epsilon RI signalling pathway, ABC transporters, mitogen-activated protein kinase (MAPK) signalling pathway, and apoptosis. As shown in Fig. 5c, ENSMUST00000129245 (Meg3) correlated with 169 protein-coding genes, enriched to dozens of KEGG pathway, such as gap junction, ABC transporters, peroxisome, glutathione metabolism, GnRH signalling pathway, complement and coagulation cascades, peroxisome proliferator-activated receptor (PPAR) signalling pathway, metabolism of xenobiotics by cytochrome P450, phagosome, tryptophan metabolism, fatty acid degradation, carbohydrate digestion and absorption, Notch signalling pathway, ribosome, and toll-like receptor signalling pathway. KEGG analysis revealed that two lncRNA transcripts were both related to ABC transporters and complement and coagulation cascades, although these two transcripts did not share the same gene of interaction. As shown in Fig. 5d, ENSMUST00000136359 (H19) correlated with 169 protein-coding genes, including vitamin digestion and absorption, galactose metabolism, carbohydrate digestion and absorption, retinol metabolism, phagosome, gap junction, arachidonic acid metabolism, butanoate metabolism, complement and coagulation cascades, antigen processing and presentation, PPAR signalling pathway, ABC transporters, Notch signalling pathway, bile secretion, and MAPK signalling pathway.

### Cis- and tans-regulator of DELs

To better understand the functions of differentially expressed lncRNAs in tx-j mice, the functions of cis- and trans-target genes of the differentially expressed lncRNAs were predicted, and then lncRNA-mRNA pairs that had a PCC greater than 0.98 (or less than − 0.98) and p-value < 0.01 were selected. It is apparent that there are 37 cis-regulatory genes of 42 lncRNAs. In addition, the 41 lncRNAs have 47 trans-regulatory genes, filtered by Blastn and RNAplex (Table 3). Five pairs of lncRNA-mRNA (ENSMUST00000156612/Apoa4, NONMMUT143909.1/C9, ENSMUST00000206226/Cyp2a4, MSTRG.62181.1/Gsta1, and MSTRG.41324.1/Mup11) presented regulatory characteristics of both cis- and trans-. There are some DELs target genes involved in signal pathways that have been identified as playing a role in liver injury induced by tx-j mice, such as ABC transporters (ABCC5, ABCC9), chemokine signalling pathway (CCR5), ECM-receptor interaction (Sdc4), and complement and coagulation cascades (C9).

**Table 3:** Top10 connections of up- and down-regulated DELs and protein-coding genes in lncRNA-mRNA coexpression network.

GENES	Degee	Updown	Type	GENES	Degee	updown	Type
MSTRG.17166.1	430	UP	lncRNA	Cd84	87	UP	Protein-coding
MSTRG.65889.1	415	UP	lncRNA	Lipn	86	UP	Protein-coding
NONMMUT154173.1	374	UP	lncRNA	Cln6	84	UP	Protein-coding
MSTRG.65372.1	371	UP	lncRNA	Muc13	84	UP	Protein-coding
MSTRG.66158.2	369	UP	lncRNA	Serpina5	83	UP	Protein-coding
MSTRG.65819.1	365	UP	lncRNA	Bmp8b	82	UP	Protein-coding
NONMMUT015735.2	364	UP	lncRNA	Gypa	82	UP	Protein-coding
NONMMUT040581.2	362	UP	lncRNA	Rbp2	82	UP	Protein-coding
MSTRG.5191.5	359	UP	lncRNA	Tmem45b	82	UP	Protein-coding
NONMMUT025830.2	357	UP	lncRNA	Rab31	81	UP	Protein-coding
NONMMUT149595.1	222	DOWN	lncRNA	Aadac	71	DOWN	Protein-coding
NONMMUT099727.1	175	DOWN	lncRNA	Sardh	67	DOWN	Protein-coding
NONMMUT143057.1	156	DOWN	lncRNA	Urad	66	DOWN	Protein-coding
MSTRG.22013.1	149	DOWN	lncRNA	C6	65	DOWN	Protein-coding
NONMMUT062890.2	140	DOWN	lncRNA	C8g	62	DOWN	Protein-coding
NONMMUT099726.1	138	DOWN	lncRNA	Ugt3a2	62	DOWN	Protein-coding
ENSMUST00000156693	122	DOWN	lncRNA	Apon	54	DOWN	Protein-coding
ENSMUST00000142299	121	DOWN	lncRNA	Cyp4v3	52	DOWN	Protein-coding
NONMMUT054921.2	103	DOWN	lncRNA	Sdr9c7	51	DOWN	Protein-coding
NONMMUT059036.2	99	DOWN	lncRNA	C4bp	46	DOWN	Protein-coding

### Real-time quantitative PCR validation

Validation of expression of DELs by RT-qPCR. Data presented from Table 1, ENSMUST00000129245, ENSMUST00000150851, ENSMUST00000136359, and ENSMUST00000181536 were identified to be significantly up-regulated in the TX group compared to the control (P < 0.01), consistent with the RNA-seq data.NONMMUT149595.1 and NONMMUT099727.1 were significantly down-regulated in the TX group compared to the control (P < 0.01), also consistent with the RNA-seq data. The results of RNA-seq were consistent with those of RT-qPCR.In a few words, the reliability of RNA-seq results was verified.

## Discussion

Even though lncRNA, once mistaken for gene transcription "dark matter", rarely has protein-coding functions, it has been found to be heavily involved in important biological functions, such as regulating cell proliferation and differentiation, the cell cycle, and apoptosis^8^, especially in organ fibres and tumours^9–13^.

In this study, the differential lncRNA expression profiles and co-expression network of lncRNA-mRNA inTX mice were assessed by deep data analysis. Our results identified 3616 lncRNA transcripts in the control and model groups. Among these, 2564 up-regulated and 1052 down-regulated lncRNAs were identified by fold change > 2 and P < 0.05. Go analysis of the co-expression network of lncRNA-mRNA showed key involvement in leukocyte transendothelial migration, glutathione metabolism, regulation of actin cytoskeleton, natural killer cell-mediated cytotoxicity, Fc epsilon RI signalling pathway, cell adhesion molecules, apoptosis,chemokine signalling pathway (CCR5), ECM-receptor interaction (Sdc4),complement and coagulation cascades (C9),and ABC transporters(ABCC5, ABCC9). KEGG analysis revealed enrichment in the drug metabolism-cytochrome P450 pathway, chemokine signalling pathway, p53 signalling pathway, toll-like receptor signalling pathway, Notch signalling pathway, PPAR signalling pathway, and MAPK signalling pathway.

In summary, WD is a complex process involving many lncRNAs, mRNAs, and pathways. lncRNAs ENSMUST00000129245, ENSMUST00000150851, ENSMUST00000136359, and ENSMUST00000181536 were differentially expressed and might play major roles in the development of WD. Key genes, for instance,Meg3,H19,and Snhg18,may be key biomarkers for WD.

MEG3 is an imprinted gene located at 14 q32, which encodes a lncRNA associated with multiple human cancers. It has been found that MEG3 expression decreased during liver fibrosis, and increased expression could activate p53 and induce apoptosis through the mitochondrial pathway^14^, suggesting that MEG3 plays an important role in HSC activation and liver fibrosis^14,15^. lncRNA H19 has been shown to play a positive role in HSC activation and proliferation, and is closely linked to liver fibrosis^16,17^. A previous study showed that decreased expression of lncRNA-H19 inhibited HSC activation and alleviated liver fibrosis in vivo and in vitro^18^. lncRNA SNHG18 acts as a tumour suppressor in hepatocellular carcinoma(HCC)and an independent diagnostic marker for liver cancer^19^; it promots cell motility by regulating EMT progression and remodelling the cytoskeleton^20^. The clinical and diagnostic value of SNHG18 in patients with HCC was investigated for the first time, and it was found that SNHG18 was significantly down-regulated in HCC tissues compared to the corresponding noncancerous tissues^21^. These may be the main target genes of IncRNAs, in WD liver fibrosis. which may compete with miRNAs. Identifying additional lncRNAs associated with liver fibrosis and further exploring their function is important for liver fibrosis intervention strategies.

PPAR is a ligand-activated receptor body, and recent studies have shown that PPAR-γ inhibits HSC activation, proliferation, and ECM formation^22^, and is closely related to liver fibrosis. The Notch signalling pathway has been confirmed to be closely related to HSC activation. Studies have shown that the Notch1 and TGFß/BMP signalling pathways can regulate gene expression of Hesl and thus induce HSC activation, suggesting that Notch signalling pathways regulate the activation of HSCs involved in the progression of liver fibrosis. Furthermore, a high expression of Notch3 is positively correlated with the activation of HSCs^23^. The drug metabolism-cytochrome P450 pathway is a group of mixed functional oxidase systems on the smooth endoplasmic reticulum, the most important enzymes of liver metabolism, and closely related to oxidative stress^24^. Excess reactive oxygen species production can enhance lipid peroxidation and damage cell biofilms, which through synergistic cytokines and hepatocyte apoptosis, contribute to hepatocellular inflammation, necrosis, and fibrosis^25^. The MAPK signal transduction pathway, ERK1/2 signal transduction pathway, JNK, and p38MAPK signal transduction pathways are significant in liver inflammation and apoptosis, and can affect the formation of liver fibrosis by regulating HSC activation, proliferation, and apoptosis^26^. Overall, the lncRNA-mRNA co-expression network was remarkably enriched in the PPAR signalling pathway, Notch signalling pathways, drug metabolism-cytochrome P450 pathway, and MAPK signalling pathway; these signalling pathways might play major roles in the pathogenesis and development of WD liver injury.

While further experiments are required, this study, nonetheless, contributes vital information regarding the molecular mechanisms of WD. This study provides a foundation for the development of new diagnostic markers and therapeutic targets for clinical treatment of WD. This study provides a new diagnostic index and treatment target for clinical treatment of WD.

## Material and methods

### Ethics statement

Seven pairs of TX mice were obtained from the USA Jackson Experiment Centre. This study protocol was approved by the Committee on the Ethics of Animal Experiments of Anhui University of Chinese Medicine (Permit Number: AHAU2018008).

### Animal experiments and sample collection

TX mice were selected as study subjects as they are the most similar animal model to humans for studying hepatolenticular degeneration, with 82% sequence homology of the ATP7B gene^27^. These mice exhibit biochemical, pathological, and clinical symptoms similar to hepatolenticular degeneration in humans.

All the TX mice were permitted free access to food and water, and lived alone under standard conditions (18–22 °C and 40–60% humidity). After 1 week acclimatisation, the mice were randomly assigned to two groups (n = 2), namely the control and model groups.

Female and male mice of 8 to 10 weeks of age and TX mice (20 ± 2G) and DL mice were obtained from the Jackson Experimental Animal Center of America. This study was rigorously carried out on the recommendation of Guide for the Care and Use of Laboratory Animals of the National Institutes of Health. Animals use protocol was reviewed and approved by the Institutional Animal Care and Use Committee of Anhui hospital of TCM.

In Wilson and control group, oxygen was supplied independently in isolation cage, and food and water were obtained freely, under an alternating 12-h light/dark cycle for 4 weeks.

After the 4^th^ week, the mice in each group were subjected to fasting for 12 h, and an aesthetized intraperitoneally with sodium pentobarbital (2 mL/kg; Shanghai Chemical Reagent Company). Hepatic tissues were harvested.One section was added with 4% paraformaldehyde for 3 h, dehydrated with ethanol and xylene, embedded in paraffin, and sliced for pathological analysis. Another liver section was sub-packaged, sealed in freezing tubes, and stored at − 80 °C.

### RNA-seq

Library construction and RNA-seq were performed by Shanghai OG Company (Ao-Ji Biotech, Shanghai, China). In brief, the total RNA of each sample was readied using an RNeasy Mini Kit (QIAGEN, Germany). Libraries were created as per the benchmark TruSeq protocol. Purified cDNA libraries were used for cluster generation and sequenced on the Illumina HiSeq 2500 according to the manufacturer's protocol.

### lncRNAs annotation and differentially expressed lncRNAs identification pipeline

After masking the adaptor sequences and the contaminated reads were removed, pure reads were processed for in silico analysis. The reads were mapped using TopHat, with 2 mismatches allowed. The expression of RNA in the liver was expressed in FPKM, and calculated using the TopHat and Cufflinks packages^28^. Transcripts with class code “i,” “r,” “u,” “x,” and “.” were selected as novel long transcripts. New transcripts were compared to other annotation databases including NONCODE (v4) (http://www.noncode.org), NCBI RefSeq, UCSC, and Ensembl^29,30^. CPAT (v1.22)^31^ was used to estimate the coding potential of each novel transcript. Transcripts with a CPAT score < 0.487 were considered to lack coding potential, and were subjected to a BLASTX search for similar protein sequences. In brief, 10,000 mRNA sequences and 10,000 subsequences included in the random selection were used as a training dataset to evaluate a mouse-specific cut-off CAPT score by comparing Ensembl coding genes by AUC analysis. Because it is the maximum sensitivity and specificity threshold, a cut-off value of 0.487 was selected. The department of operations not documented in BLASTX is considered the new lncRNA. After the lncRNAs were identified and quantitation performed, classification was conducted based on the location between lncRNAs and mRNAs^32^. Furthermore, chromosome information was also annotated.

### The lncRNA-mRNA-co-expression network

Co-expression networks of lncRNA-mRNA are typically used to analyse the functional and regulatory involvement of lncRNAs. Functionally related lncRNAs are expected to be associated with functionally similar mRNAs. To identify the interactions between lncRNAs and mRNAs, we constructed a gene co-expression network according to the normalised FPKM of unit genes^33^. After screening the data for differentially expressed lncRNAs and mRNAs, PCC between lncRNAs and mRNAs was calculated and retained a pair(only lncRNA-mRNA) of significant correlations (PCC > 0.98 and P < 0.05)^34^. The nodes degree was calculated to examine the topological property of this schematic, which was defined as the number of directly linked neighbours. The function of four hub-lncRNAs, which have high degrees of expression, were assessed by GO and KEGG pathways terms that are enriched in co-expressed protein-coding genes of each lncRNA.

### Prediction of cis- and trans-target genes

Predicting potential targets of lncRNAs, the algorithms of Cis- or trans-acting algorithms were often credible. Based on the chromosomal location by using genome browser, cis-acting potential target genes should be physically located within 10 kb upstream or 10 kb downstream of lncRNAs. The trans-acting potential target genes of lncRNAs were predicted based on the lncRNA-mRNA sequence complementary and predicted lncRNA-mRNA duplex energy. In brief, BLASTN was performed to survey mRNA sequences with identity > 95% and E-value < 1E − 5 and the RNAplex software was ued to calculate the duplex energy with RNAplex-E-30. Pearson's correlation coefficients were calculated with the expression of lncRNAs and mRNAs. Cluster Profiler was ued to analyse the enrichment function of the lncRNA target genes, and P < 0.05 was considered significant.

### RT-PCR validation

RNA-seq results were validated by RT-PCR, and six lncRNAs (ENSMUST00000129245, ENSMUST00000150851, ENSMUST00000136359, ENSMUST00000181536, NONMMUT149595.1, and NONMMUT099727.1) were selected for qPCR validation. cDNA was synthesised by reverse transcription of RNA from two groups of mouse liver tissues as templates, using 2-CT relative determination quantitative analysis, with beta-actin as an endogenous control. To detect the non-processing group for the calibration sample of lncRNA transfer in the sample recorded level, each sample was calculated by taking the mean of 3 tests in parallel. The primers used are listed in Table 1.

### Statistical analysis

Quantitative data of qPCR are presented as means ± SD. Statistic Package for Social Science 22.0 software (SPSS, Chicago, USA) was employed for statistical analysis using the Student’s t-test and threshold statistical significance value of p < 0.05. Using the Hmisc package in R, the PCC between lncRNAs and mRNAs was calculated based on the expression determined using RNA-seq FPKM (PCC > 0.95, P < 0.05).

## Data Availability

The datasets generated during and/or analysed during the current study are available from the corresponding author on reasonable request.
